# Dataset for mosquitoes (Diptera: Culicidae) from Vaca Key, Monroe County, Florida USA

**DOI:** 10.3897/BDJ.8.e55059

**Published:** 2020-07-27

**Authors:** Lawrence J. Hribar

**Affiliations:** 1 Florida Keys Mosquito Control District, Florida, United States of America Florida Keys Mosquito Control District Florida United States of America

**Keywords:** Diptera, Culicidae, seasonal distribution, species composition, relative abundance

## Abstract

**Background:**

The Florida Keys Mosquito Control District has used dry ice-baited light traps to monitor mosquito populations on Vaca Key since 1998. The first site sampled was monitored continuously for almost 20 years until all vegetation was removed.

**New information:**

This paper describes a dataset compiled over almost 20 years of continuous trapping along Manor Lane on Vaca Key, Florida.

## Introduction

### Background

The Florida Keys Mosquito Control District conducted adult mosquito surveillance along Manor Lane in Vaca Key for almost twenty years. Surveillance was accomplished by use of dry ice-baited light traps. Traps were set and retrieved weekly except for interruptions due to unavoidable situations, such as storms and illness. Traps were provisioned with 2 pounds (ca. 1 kg) of dry ice, deployed in the late afternoon and retrieved the following morning. Traps were hung from the same tree limb each time they were set. Mosquitoes were returned to the laboratory, killed by freezing and identified to species. This paper reports data pertaining to 32 mosquito species.

## General description

### Purpose

These data were collected to document the species composition, seasonal distribution and relative abundance of mosquitoes on Vaca Key, Florida.

## Sampling methods

### Study extent

Monitoring of the mosquito fauna on Vaca Key began in the late summer of 1998 and continued until the winter of 2017. Collections were intended to be made weekly although, due to storms, illness and vacations, this was not always possible.

### Sampling description

A battery-powered light trap (American Biophysics Company, Clarke, John Hock) was baited with approximately two pounds (ca. 1 kg) of dry ice and hung from the same tree limb once per week for over 19 years. The trap was deployed in the late afternoon and retreived the following morning. The trap collection was taken to the laboratory, frozen, and all mosquitoes separated, identified, and counted. Data were recorded in spreadsheets. Voucher specimens of all taxa are retained in the synoptic mosquito reference collection maintained by the Florida Keys Mosquito Control District. Data may be found in Suppl. material 1.

## Geographic coverage

### Description

The trap was deployed on Vaca Key, Monroe County, Florida, USA. The coordinates of the trap location trap were: 24°42'78"N, 81°04'56"W.

## Taxonomic coverage

### Description

Thirty-two mosquito species were documented during the sampling period.

### Taxa included

**Table:** 

Rank	Scientific Name	Common Name
species	*Aedes taeniorhynchus*	Black salt marsh mosquito
species	*Deinocerites cancer*	Crabhole mosquito
species	*Culex nigripalpus*	
species	*Anopheles atropos*	
species	*Culex quinquefsciatus*	Southern house mosquito
species	*Anopheles crucians*	
species	*Culex bahamensis*	
species	*Aedes infirmatus*	
species	*Aedes atlanticus*	
species	*Psorophora ciliata*	
species	*Psorophora columbiae*	
species	*Aedes sollicitans*	Saltmarsh mosquito
species	*Aedes aegypti*	Yellowfever mosquito
species	*Aedes triseriatus*	Eastern treehole mosquito
species	*Uranotaenia lowii*	
species	*Anopheles quadrimaculatus*	Common malaria mosquito
species	*Culex erraticus*	
species	*Culex atratus*	
species	*Culex iolambdis*	
species	*Culex peccator*	
subgenus	Culex (Melanoconion)	Unidentified *Melanoconion*
species	*Culex salinarius*	
species	*Culiseta melanura*	
species	*Wyeomyia mitchellii*	
species	*Psorophora ferox*	
species	*Psorophora johnstonii*	
species	*Aedes tortilis*	
species	*Anopheles albimanus*	
species	*Aedes condolescens*	
species	*Culex declarator*	
family	Culicidae	Other unidentified
species	*Anopheles grabhamii*	
species	*Culiseta inorrnata*	
species	*Aedes albopictus*	Asian tiger mosquito

## Traits coverage

The study site was described by Hribar (2002). Hribar et al. (2018) described population declines for the four most commmonly collected species

## Temporal coverage

### Notes

Data collection began on 17 August 1998 and ended on 27 December 2017.

## Usage rights

### Use license

Creative Commons Public Domain Waiver (CC-Zero)

## Data resources

### Data package title

CSV

### Number of data sets

1

### Data set 1.

#### Data set name

Manor Lane BDJ

#### Data format

Data in spreadsheet, csv.

#### Number of columns

38

#### Description

Number of adult female mosquitoes collected per trap per night. NA indicates that the species was not reported and so no numbers are available.

**Data set 1.** 

Column label	Column description
Year	Year of Collection
DOY	Day of Year: Ordinal date of collection; 1-365, 1-366 for leap years
Date	Date of collection, ISO 8601 forrmat (YYYY-MM-DD)
Season	Season of collection, based on equinoxes and solstices: W, winter; S, spring; U, summer; F, fall (autumn)
Latitude	Trap location coordinate; degrees, minutes, seconds
Longitude	Trap location coordinate; degrees, minutes, seconds
Aeae	*Aedes aegypti*
Aeal	*Aedes albopictus*
Aeat	*Aedes atlanticus*
Aeco	*Aedes condolescens*
Aein	*Aedes infirmatus*
Aeso	*Aedes sollicitans*
Aeta	*Aedes taeniorhynchus*
Aetr	*Aedes triseriatus*
Anal	*Anopheles albimanus*
Ancr	*Anopheles crucians*
Angr	*Anopheles grabhamii*
Anqu	*Anopheles quadrimaculatus*
Csin	*Culiseta inornata*
Csme	*Culiseta melanura*
Cuat	*Culex atratus*
Cuba	*Culex bahamensis*
Cude	*Culex declarator*
Cuer	*Culex erraticus*
Cuio	*Culex iolambdis*
Cuni	*Culex nigripalpus*
Cupe	*Culex peccator*
Cuqu	*Culex quinquefasciatus*
Cusa	*Culex salinarius*
Deca	*Deinocerites cancer*
Psci	*Psorophora ciliata*
Psco	*Psorophora columbiae*
Psfe	*Psorophora ferox*
Psjo	*Psorophora johnstonii*
Urlo	*Uranotaenia lowii*
Wymi	*Wyeomyia mitchellii*
UINM	Unidentified specimens (not Culex (Melanoconion))
UnCM	Unidentfied Culex (Melanoconion) specimens

## Supplementary Material

9A80E46B-7502-5194-B548-621A6EECD0C2 10.3897/BDJ.8.e55059.suppl1 Supplementary material 1 Manor Lane BDJ Data type Count data in a csv spreadsheet. Brief description Approximately 19.5 years of near-weekly mosquito collections from Vaca Key, Monroe County, Florida. File: oo_388380.csv https://binary.pensoft.net/file/388380 Hribar, L.J.
